# Insomnia

**Published:** 1905-07-22

**Authors:** 


### INSOMNIA.

Dr. Arthur Foxwell,[1] in a short communication on this important subject draws attention to the readiness with which patients and their advisers fly to the use of narcotic drugs. He considers that abnormal conditions of the cerebral circulation are the fundamental factors in the production of insomnia. Although Gulland and others have demonstrated the existence of peri-vascular nerve plexuses on the cerebral vessels, yet experimental physiology points to the conclusion that these have very little effect on the circulation in the brain. There seems little doubt that the circulatory conditions in the brain are governed by the action of the vaso-motor system generally, that is to say by the arterial blood-pressure in the aorta.

He states that clinically both high and low tensions of the blood are competent to cause insomnia. High tension tends to raise the intracranial pressure. The first effect of this is to displace some of the cerebro-spinal fluid, but this stage is soon past, and the next phase is an increase in the intra-vascular tension in the brain, which both increases the quantity of blood passing through the cerebral vessels in a given time, and also increases the velocity of its flow. Both these factors necessarily lead to greater activity of the brain-cells, and thus to insomnia. The author quotes as instances confirmatory of his thesis, the case of gouty people, who are often intellectually active and from time to time suffer from sleeplessness, while the tension in their arteries is generally above the average. At the same time, persistent and unvarying high tension, unless it be extreme, does not cause any divergence from the normal as regards sleep. It is the suddenness of a change in arterial pressure which disturbs the equilibrium, and if that change be in the direction of a rise of tension, the logical antidote lies in measures which help to reduce the exaggerated tension. If this can be accomplished, the author considers that sleep will be restored, but not immediately, perhaps, since the excited brain-cells require time to recover their equanimity.

Dr. Foxwell thinks that in daily life the commonest cause of sudden temporary rises in blood-pressure is the imperfect assimilation of food, with a resultant contraction of the splanchnic vessels. His method of procedure, therefore, is the administration of stomachics, such for instance as the following pill:—

| | |
|---|---|
| Pulv. Rhei. ... ... ... ... | 1 gr. |
| Hyd. Subchlor. ... ... ... | $\frac{1}{15}$ gr. |
| Pulv. Zingib. ... ... ... | $\frac{1}{4}$ gr. |
| Ext. Hyoscyami. ... ... ... | $\frac{1}{2}$ gr. |

Fiat pillula.

Sig. One to be taken four times a day after food.

On the other hand, insomnia is commonly produced by the excessive fall in blood-pressure consequent upon great fatigue. In such cases, of course, the indications for treatment are to be sought in the opposite direction. Thus the author states that in cases of this kind a cup of tea is often a good hypnotic, or a hypodermic injection of strychnine. If at the same time as the bodily vitality is thus depressed there exist signs that the brain is in a state of irritable activity, it is recommended that the stimulant should be combined with a moderate dose of bromide of potash, or veronal in doses of 3 to 6 grs.

[1] Birm. Med. Rev., May 1905.

